# Spatial Heterogeneity of Gut Microbial Composition along the Gastrointestinal Tract in Natural Populations of House Mice

**DOI:** 10.1371/journal.pone.0163720

**Published:** 2016-09-26

**Authors:** Taichi A. Suzuki, Michael W. Nachman

**Affiliations:** Department of Integrative Biology, Museum of Vertebrate Zoology, University of California, Berkeley, California, United States of America; Wageningen Universiteit, NETHERLANDS

## Abstract

There is a growing appreciation of the role of gut microbial communities in host biology. However, the nature of variation in microbial communities among different segments of the gastrointestinal (GI) tract is not well understood. Here, we describe microbial communities from ten different segments of the GI tract (mouth, esophagus, stomach, duodenum, ileum, proximal cecum, distal cecum, colon, rectum, and feces) in wild house mice using 16S rRNA gene amplicon sequencing. We also measured carbon and nitrogen stable isotopic ratios from hair samples of individual mice as a proxy for diet. We identified factors that may explain differences in microbial composition among gut segments, and we tested for differences among individual mice in the composition of the microbiota. Consistent with previous studies, the lower GI tract was characterized by a greater relative abundance of anaerobic bacteria and greater microbial diversity relative to the upper GI tract. The upper and lower GI tracts also differed in the relative abundances of predicted microbial gene functions, including those involved in metabolic pathways. However, when the upper and lower GI tracts were considered separately, gut microbial composition was associated with individual mice. Finally, microbial communities derived from fecal samples were similar to those derived from the lower GI tract of their respective hosts, supporting the utility of fecal sampling for studying the gut microbiota of mice. These results show that while there is substantial heterogeneity among segments of the GI tract, individual hosts play a significant role in structuring microbial communities within particular segments of the GI tract.

## Introduction

Recent advances in microbial ecology have demonstrated important roles of gut microbes in digestion [1], immunity [2], development [3], and behavior [4] of hosts. Despite the importance of gut microbial communities in host biology, many studies depend solely on fecal samples to investigate the gut microbial community. Less attention has been given to spatial heterogeneity along the gastrointestinal (GI) tract or to the mechanisms structuring microbial variation along the GI tract.

In mammals, diet seems to be a major driver of fecal microbial communities within [5] and between species [6, 7]. However, hindgut and foregut fermenters show distinct fecal microbial communities despite eating similar herbivorous diets [6, 7]. Hindgut fermenters have a simple stomach, and the majority of fermentation takes place in the enlarged cecum. In contrast, foregut fermenters have, in addition to the cecum, a segmented stomach where the majority of fermentation takes place. This suggests that fecal microbial communities could be partly determined by gut anatomy [6] along with other factors including host genotype [8] and geography [9].

One approach to understanding the factors affecting the gut microbiota along the GI tract is to assess the relative importance of gut segments and individual hosts in explaining the composition of the microbial community. For example, if variation among gut segments explains most of the variation in microbial composition, this might suggest that gut-segment-specific biochemical differences (e.g. pH, oxygen, nutrients, etc.) determine microbial communities along the GI tract [10–14]. In contrast, if variation among individuals explains most of the microbial variation, this would suggest that differences in diet, host genotype, and/or host habitat are important in determining the composition of microbial communities [15, 16]. The two hypotheses above are not mutually exclusive.

Characterizing the complete GI tract is important not only for understanding microbial composition in different gut segments but also for assessing the efficacy of fecal sampling for studying gut microbial communities. The assumption that fecal microbial communities are similar to those in the colon has been examined mostly in humans, and the two types of samples usually show significant differences [10, 11, 15, 17, 18]. Recently, multiple sites along the GI tract have also been characterized in laboratory mice [12], wood rats [13], flying squirrels [14], and rhesus macaques [19]. For example, in wood rats, the fecal microbial community is different from that in the large intestine and is more similar to the communities found in the stomach or small intestine [13]. In contrast, in rhesus macaques, the relative abundances of bacterial taxa in fecal samples were significantly correlated with those in both the large and small intestines [19]. Together, these studies suggest that fecal microbial communities may be most similar to different parts of the GI tract in different host species.

Wild house mice provide a useful mammalian model for studying microbial variation along the GI tract. Laboratory strains of house mice have been used extensively as a model in gut microbial ecology. Microbial variation along the GI tract has been characterized in one strain of inbred mice in a laboratory environment [12]. The authors sampled seven locations along the GI tract in six individuals of a single genotype, and found differences in the microbial community mainly between the upper and lower GI tract [12]. Wild mice are genetically variable, and host genotype is known to play an important role in shaping the gut microbiota [20]. Wild mice may also show variation in microbial communities due to differences among animals sampled from different localities [21, 22]. In addition, laboratory diets and environment have been shown to alter the gut micobiota in *Drosophila* [23] and house mice [24–26] compared to their wild relatives. Finally, house mice live in close association with humans. The parallel ecology between humans and house mice highlights the importance of studying the microbial composition of wild house mice for translational research [27, 28].

Here, we collected wild house mice in Tucson, Arizona. First we describe the microbial heterogeneity along ten segments of the GI tract. We then assess the relative importance of gut segment, host individual, and diet in explaining the composition of the microbial communities observed in our samples.

## Materials and Methods

### Animal and sample collections

Three adult male and three adult female *Mus musculus* were collected from Tucson, Arizona on September 19, 2012 (Table A in S1 File). Animals were collected on private property with the permission of the landowners under a State of Arizona scientific collecting permit (LIC# SP791101 to Taichi Suzuki). Sherman live traps were used without bait to assess the “natural” gut microbial composition. Animals were collected from four localities (Table A in S1 File). Animals were kept in Sherman traps, euthanized by cervical dislocation, and all samples were collected within 24 hours after capture. All procedures involving animals were reviewed and approved by the University of Arizona Institutional Animal Care and Use Committee (protocol 07–004). Museum specimens (skins and skulls) were prepared and have been deposited in the mammal collection of the Museum of Vertebrate Zoology at the University of California, Berkeley (catalog numbers MVZ230525, 230526, 230530, 230535, 230537, and 230539).

**Fig 1:**
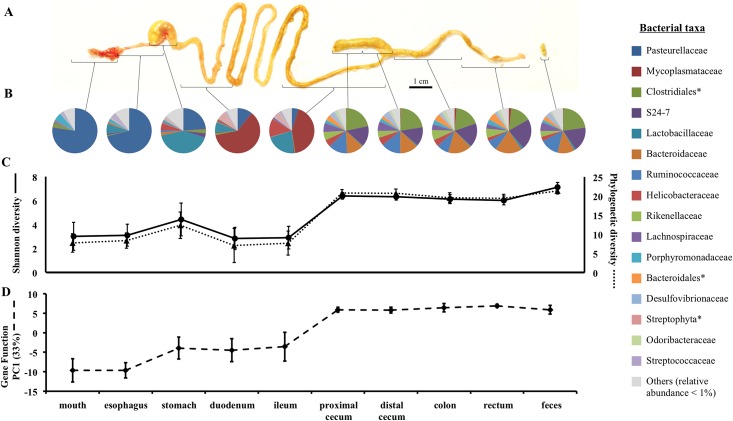
Spatial heterogeneity of microbial composition along the gastrointestinal (GI) tract. Ten samples were collected along the GI tract per individual (A). The averages of relative abundance of bacterial families (B), the microbial diversity measures (C), and the first principle component value from predicted gene function categories (D) across the GI tract are shown. The error bars are standard deviations. Asterisks denote unclassified families within the listed order.

The samples were collected from the following 10 locations along the GI tract under sterile conditions: oral cavity, esophagus, stomach, duodenum, ileum, proximal half of cecum, distal half of cecum, proximal colon, rectum, and fresh feces (Fig 1A). The oral cavity was swabbed using 1 cm^2^ kimwipe. All tissues were collected immediately after the animal was euthanized. All samples were stored immediately at -80°C.

### Stable isotope analyses

Hair samples were used to infer diet based on carbon and nitrogen stable isotope ratios. Hair samples (0.4 mg) were collected from the base of the right hind leg from each individual. The hair was rinsed in 2:1 chloroform:methanol to remove the surface oils, rinsed in distilled water, and dried in collection tubes. Stable isotope analyses were performed in the Environmental Isotope Laboratory at the University of Arizona. Carbon (δ^13^C) and nitrogen (δ^15^N) stable isotope ratios were measured on a continuous-flow gas-ratio mass spectrometer (Finnigan Delta PlusXL), which was coupled to an elemental analyzer (Costech). Standardization was based on acetanilide for elemental concentration (δ^13^C: NBS-22 and USGS-24, δ^15^N: IAEA-N-1 and IAEA-N-2).

### DNA extraction and Sequencing

The frozen samples were chopped into pieces with a sterile razor blade in a petri dish on dry ice. We used 200 mg of each sample, except for those from the mouth and esophagus which were smaller. To study microbes living in the gut contents and on the mucosal surface, the entire sample with the host tissue was immediately disrupted and rinsed with sterile forceps in 1.4ml ASL (from QIAamp DNA stool Minikit). Most of the host tissue was removed by this procedure. Following a protocol developed by Smith et al. (2011) to extract microbial DNA [29], 0.2 g of sterile zirconia/silica disruption beads (0.1mm, Research Products International Corp.) were added and vortexed until the samples were thoroughly homogenized. Samples were then placed in a TissueLyser LT (Qiagen) 30HZ for 6 min for further mechanical disruption. The suspension was heated at 95°C for 5 min, and DNA was extracted following step 4 (vortex and centrifugation) in the protocol from the QIAamp DNA stool Minikit (Qiagen). The quality of the DNA was evaluated based on Nano Drop 3300 (Thermo Scientific). DNA samples were stored at -20°C before sequencing.

The DNA samples were shipped to Argonne National Laboratory for 16S rRNA amplicon sequencing at their Next Generation Sequencing Core Facility. The V4 region of the 16S rRNA gene was amplified and the samples were multiplexed for sequencing on a 150bp paired-end Illumina MiSeq platform using primers 515F (5’—GTGCCAGCMGCCGCGGTAA) 806R (5’—GGACTACHVGGGTWTCTAAT) and barcodes described in [30]. All data have been deposited into the European Nucleotide Archive (accession number PRJEB15238).

### Analyses

The paired-end reads were merged in PANDAseq [31] and all the merged sequences were analyzed in QIIME version 1.8.0 [32]. Sequences were demultiplexed and quality-filtered using the default parameters in QIIME. Chimeric sequences were detected and removed by following both the reference (Greengenes 13_8) [33] and de-novo based approaches using USEARCH/UCHIME 6.1 [34, 35]. One sample (TAS203.7, Table A in S1 File) was excluded from all analyses due to low sequence reads. We assigned sequences to OTUs by using the subsampling open-reference approach with default parameters in QIIME [36]. Briefly, sequences were initially clustered against a reference database (Greengenes 13_8) [33] using UCLUST [34] with a minimum sequence identity of 97%. A random subsample of the reads that fail to hit the reference database was subsequently clustered de novo with default parameters. Singleton OTUs were removed. Taxonomy was assigned using uclust-based consensus taxonomy with default parameters. A phylogenetic tree was created from representatives of all OTUs using FastTree [37]. The OTU table was rarefied by random sampling (without replacement) at an even depth of 2000 reads to maximize the samples included in the analyses. On average, a total of 16,026 merged pair-end reads per sample were obtained after default quality filtering and chimera removal. The median amplicon length was 253bp after merging. These sequences resulted in a total of 7,137 OTUs. The major conclusions hold at different sequencing depths (Figure A in S1 File). Detailed information on the number of reads per sample is provided in Table A in S1 File.

The relative abundances of bacterial families and several measures of diversity were calculated using the rarefied OTU table. The number of OTUs, Shannon diversity index, and Faith’s phylogenetic diversity [38] were calculated. The number of OTUs provides a measure of species richness, while Shannon’s diversity index provides an integrative measure of evenness and richness, and Faith’s phylogenetic diversity is based on the cumulative phylogenetic branch lengths. For each sample, we calculated the mean of 20 iterations based on a subsampling of 2000 sequences. The differences in relative abundances of bacterial taxa and microbial diversity between the gut segments were tested using Kruskal-Wallis tests and Wilcoxon rank sum tests with Bonferroni corrections in JMP 12.1 (SAS institute). A two-tailed significance threshold of 0.05 was used for all the statistical tests.

To evaluate potential functional differences of the microbiome along the GI tract, gene family abundances were predicted from the 16S rRNA gene sequences using PICRUSt [39]. OTUs that are only present in the reference database (Greengenes 13_5) were included in the analyses, a requirement of PICRUSt. The OTUs were rarefied to a single depth (2000 reads) for each sample. Kyoto Encyclopedia of Genes and Genomes (KEGG) ortholog abundances were predicted for each sample, the KEGG pathway functions were categorized at level 3, and the relative abundances of functional categories were calculated. Principle component values were calculated from all of the functional categories. Kruskal-Wallis tests and Wilcoxon rank sum tests with Bonferroni corrections were used to test for differences among gut segments.

Overall similarities between microbial communities were quantified using UniFrac distances which integrate the phylogenetic information of the community [40]. Unweighted UniFrac distances (community membership; presence and absence of taxa) and weighted UniFrac distances (community structure; taking into account the relative abundances of taxa) were calculated in QIIME. To visualize the community similarity, PCoA and UPGMA hierarchical clustering analyses were conducted. Jackknife support of nodes in UPGMA trees were calculated using default settings in QIIME.

To quantify the effect size of variables explaining unweighted and weighted UniFrac distances, ADONIS was used with 999 permutations in QIIME. We also tested the effects of gut segment, individual, and diet (carbon and nitrogen stable isotopes) on gut microbial membership using Generalized Linear Models (GLMs) in R (version 3.3.0). To perform model selection, we used the Akaike information criterion with sample size correction (AICc) with the “AICc” function in the package “AICcmodavg”, as well as likelihood ratio tests in cases where models were hierarchically nested using the “lrtest” function in the package “lmtest”. We ran four separate analyses: (1) a full model, including fixed effects for gut segment, carbon, nitrogen, and individual; (2) fixed effects of carbon, nitrogen, and individual; (3) fixed effects of gut segment and individual; and (4) fixed effects of gut segment, carbon, and nitrogen. Models 2, 3, and 4 are each nested within model 1 and were compared to model 1 in likelihood ratio tests. These tests evaluate whether gut segment, diet, and individual explain significant variation in microbial communities in the context of models that include the other variables. Each of these analyses was run on each of three datasets: the complete GI data, the upper GI data, and the lower GI data, for a total of twelve analyses (see Table B in S1 File). For each dataset, the response variable was unweighted UniFrac PC1, which was calculated independently in the complete GI, the upper GI, and the lower GI. The fractions of the variation explained by PC1 were 24%, 13%, and 22%, respectively.

## Results

### Obligately anaerobic bacteria are more abundant in the lower GI tract than they are in the upper GI tract

The relative abundances of the dominant phyla showed significant differences among gut segments (Kruskal-Wallis test, Firmicutes P = 0.004, Bacteroidetes P < 0.0001, Proteobacteria P < 0.0001, Tenericutes P = 0.001, and Cyanobacteria P = 0.02). Dominant bacterial families showed a similar pattern, in which 8 out of the 10 highly abundant bacterial families displayed significant differences in relative abundance among the gut segments after Bonferroni corrections (Fig 1B and Table C in S1 File). The observed differences in the relative abundances of bacterial taxa along the GI tract were consistent with a decrease in oxygen concentration from the mouth to the anus. For example, the upper GI tract (mouth to ileum) was dominated by mostly facultatively anaerobic bacterial families such as Pasteurellaceae, Mycoplasmataceae, and Lactobacillaceae (Table D in S1 File). In contrast, the lower GI tract (cecum to feces) was dominated by mostly obligately anaerobic bacterial families (Table E in S1 File). For example, within the phylum Firmicutes, facultatively anaerobic Firmicutes (Class: Bacilli) were more abundant in the upper GI tract than in the lower GI tract (Upper GI mean: 0.21, Lower GI mean: 0.01, Wilcoxon rank sum test: P < 0.0001). In contrast, obligately anaerobic Firmicutes (Class: Clostridia) showed the opposite pattern (Upper GI mean: 0.04, Lower GI mean: 0.37, Wilcoxon rank sum test: P < 0.0001).

Greater spatial heterogeneity in the relative abundances of bacterial taxa was observed within the upper GI tract compared to the lower GI tract (Tables D and E in S1 File). For example, the relative abundances of Pasteurellaceae and Mycoplasmataceae showed significant differences among the gut segments within the upper GI tract (Kruskal-Wallis test, P < 0.005). Pasteurellaceae were more abundant in mouth, esophagus, and stomach samples (mean relative abundance = 0.57) compared to small intestine samples (mean relative abundance = 0.08) (Fig 1B and Table D in S1 File). In contrast, Mycoplasmataceae were less abundant in mouth, esophagus, and stomach samples (mean relative abundance = 0.003) and were more abundant in small intestine samples (mean relative abundance = 0.52) (Fig 1B and Table D in S1 File). However, within the lower GI tract, none of the 10 most abundant bacterial families showed significant differences in relative abundance between the gut segments (Table E in S1 File).

A total of 42 core OTUs (OTUs that are present in all individuals for each gut segment) was observed in the total dataset of 7,137 OTUs (Table F in S1 File). The number of core OTUs was greater in the lower GI tract (mean = 17.6) compared to the upper GI tract (mean = 4.8) (Wilcoxon rank sum test, P = 0.01). The distal cecum had the greatest number of core OTUs of all the gut segments. Fecal samples shared a greater fraction of core OTUs with the lower GI tract (93.3%) compared to the upper GI tract (6.7%) (Table F in S1 File).

### Microbial diversity and community phylogenetic measurements differ between upper and lower GI tract

Microbial diversity differed among the gut segments overall (Kruskal-Wallis test, P < 0.0001 for Shannon index and Phylogenetic diversity). This result was mainly driven by the significant shifts in microbial diversity between the upper and lower GI tract. The Shannon index and Phylogenetic diversity were lower in the upper GI tract compared to the lower GI tract (Fig 1C) (Wilcoxon rank sum test: P < 0.0001 for each).

Although diversity measures were calculated after rarefying the number of sequences to an equal depth to control for sampling effort, both the Shannon index and Phylogenetic diversity were significantly correlated with the number of raw sequence reads in each sample (Figure B in S1 File). In principle, this might lead to a bias in estimates of diversity. However, three lines of evidence suggest that our conclusion of lower diversity in the upper GI tract is robust. First, when we restricted the comparison to samples where the read depths were comparable between upper and lower GI tract (i.e. samples with between 11634 and 18531 reads, Figure B in S1 File), the lower GI tract had consistently higher diversity measures compared to the upper GI tract (Wilcoxon rank sum test: P < 0.0001). Second, we took the residual values between the sequence reads and the rarefied diversity measures and we compared these residuals among gut segments; the overall pattern remained the same between the upper and lower GI tract using these residual values (Wilcoxon rank sum test: P < 0.0001) (Figure C in S1 File). Finally, comparisons at different sequence depths gave a consistent pattern using the rarefaction curves (Figure A in S1 File). Therefore, we conclude that microbial diversity is greater in the lower GI tract compared to the upper GI tract.

### Predicted gene functions differ between the upper and lower GI tract

**Fig 2:**
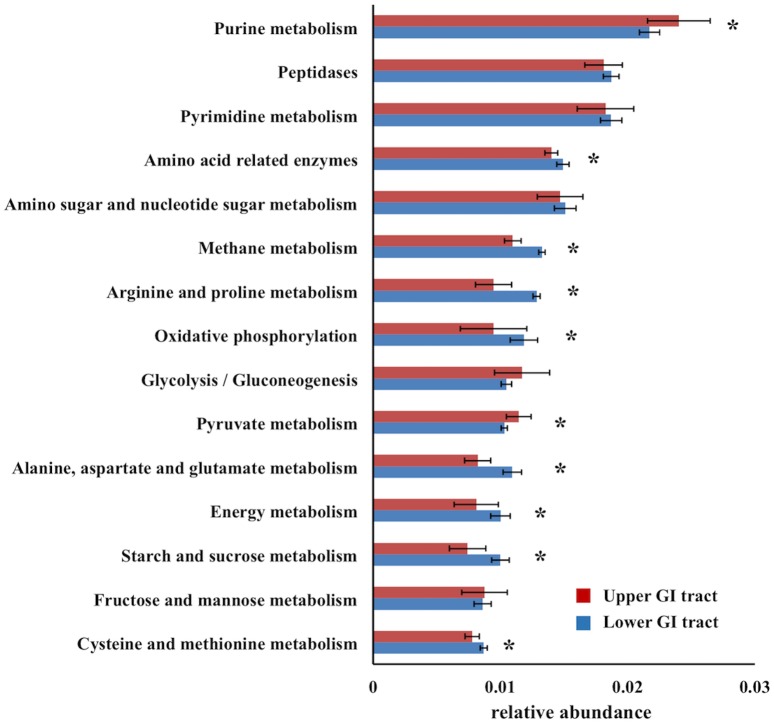
The relative proportions of the most abundant metabolism related KEGG pathways (level 3) predicted by PICRUSt between upper and lower GI tract. The error bars are standard deviations. The star indicates Bonferroni corrected P < 0.0033 using Wilcoxson rank sum test.

The predicted functions of the gut microbial community differed between the upper and lower GI tract. The first principle component of the relative abundances of microbial gene functions (KEGG pathway categories) predicted from the 16S rRNA gene data showed a significant difference between the upper and lower GI tract (Wilcoxon rank sum test P < 0.0001) (Fig 1D). The gene function PC1 was enriched in metabolism functions, where 11 out of the top 15 eigenvectors were categorized to functions related to metabolism (Table G in S1 File). Gene function PC1 significantly differed among the gut segments within the upper GI tract (Kruskal-Wallis test, P = 0.006), but not within the lower GI tract (Kruskal-Wallis test, P = 0.14). The differences among the gut segments in the upper GI tract were not significant after multiple corrections (Wilcoxon rank sum test, Bonferroni corrected P < 0.005). To test whether the abundance of genes in various metabolic pathways differed between the upper and lower GI tract, we focused on the top 15 most abundant metabolism gene function categories. Most of the metabolism gene function categories showed significant differences in their relative abundances between the upper and lower GI tract (Fig 2).

### The effects of individual host, gut segment, and diet on microbial community composition

**Fig 3:**
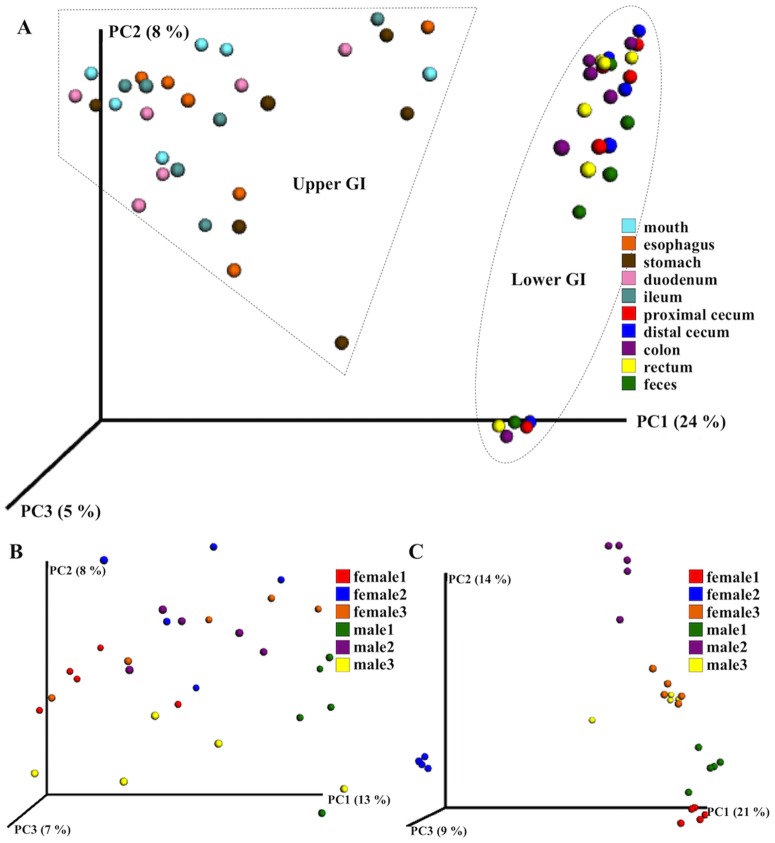
PCoA plots of microbial community membership (unweighted UniFrac distance). Each dot represents a microbial community from one gut segment in one individual. The first principle component (PC1) mostly accounts for differences between the upper and lower GI tract (A). Within the upper GI tract, microbial communities were grouped by host individual to some degree (B). Within the lower GI tract, microbial communities were strongly grouped by host individual (C).

For the complete GI tract dataset, gut segment best explained the variation in microbial community membership (R^2^ = 0.31, P < 0.001) (Fig 3A) and structure (R^2^ = 0.76, P < 0.001) based on ADONIS (Table 1). Analyses using generalized linear models (GLMs) showed a similar pattern (Table B in S1 File). The model excluding gut segment (model 2) had a significantly lower log likelihood score compared to the full model (Likelihood ratio test, P <0.001). In contrast, the model excluding diet (model 3) and the model excluding individual (model 4) were not significantly different from the full model (Table B in S1 File). This indicates that variation in diet and variation among individuals are not contributing to variation in gut microbial composition when the entire GI tract is considered. Given the large differences in microbial communities between the upper and lower GI tract, we next restricted our analyses to datasets consisting of samples from the upper GI tract alone and the lower GI tract alone.

**Table 1:** Variables explaining gut microbial communities based on ADONIS.

	Complete GI tract				Upper GI tract				Lower GI tract			
	Community membership^1^		Community structure^2^		Community membership^1^		Community structure^2^		Community membership^1^		Community structure^2^	
	R^2^	p-values	R^2^	p-values	R^2^	p-values	R^2^	p-values	R^2^	p-values	R^2^	p-values
Gut segments	**0.31**	**0.001**	**0.76**	**0.001**	0.15	0.166	**0.66**	**0.001**	0.10	0.995	0.10	0.807
Individual	**0.18**	**0.001**	0.09	0.441	**0.28**	**0.001**	0.14	0.658	**0.56**	**0.001**	**0.74**	**0.001**
Diet (δ^13^C‰)	**0.04**	**0.020**	0.03	0.192	**0.06**	**0.003**	0.06	0.182	**0.09**	**0.007**	**0.23**	**0.001**
Diet (δ^15^N‰)	**0.05**	**0.004**	0.03	0.140	**0.06**	**0.003**	0.03	0.347	**0.15**	**0.001**	**0.31**	**0.001**

^1^ Unweighted UniFrac distance, which does not depend on relative abundance.

^2^ Weighted UniFrac distance, which does depend on relative abundance.

Within the upper GI tract, gut segment was associated only with community structure (weighted UniFrac distance) (R^2^ = 0.66, P < 0.001), but not with community membership (unweighted UniFrac distance) (R^2^ = 0.15, P = 0.2) (Fig 3B and Table 1). Instead, community membership was significantly associated with host individual (R^2^ = 0.28, P < 0.001) (Table 1). The model comparison of GLMs showed a similar pattern; a model without individual (model 4) was significantly worse than the full model (Likelihood ratio test, p < 0.001). In contrast, models excluding gut segment (AICc = -19.96) or excluding diet (AICc = -12.53) were comparable to the full model (AICc = -12.53) (Table B in S1 File). Within the lower GI tract, variation in community membership and variation in community structure were both significantly associated with host individual and diet, but not with gut segments (Fig 3C and Table 1). Similarly, a model without individual (model 4) was significantly worse than the full model (Likelihood ratio test, p < 0.001) (Table B in S1 File). Models without gut segment (AICc = -94.81) or without diet (AICc = -91.61) were comparable to or better than the full model (AICc = -19.61) (Table B in S1 File). These results indicate that differences among individuals account for significant variation in the gut microbial community when the upper or lower GI tract are considered separately. Moreover, these differences among individuals explain more of the variation in the lower GI tract than in the upper GI tract (Fig 3C, Table 1, and Table B in S1 File).

**Fig 4:**
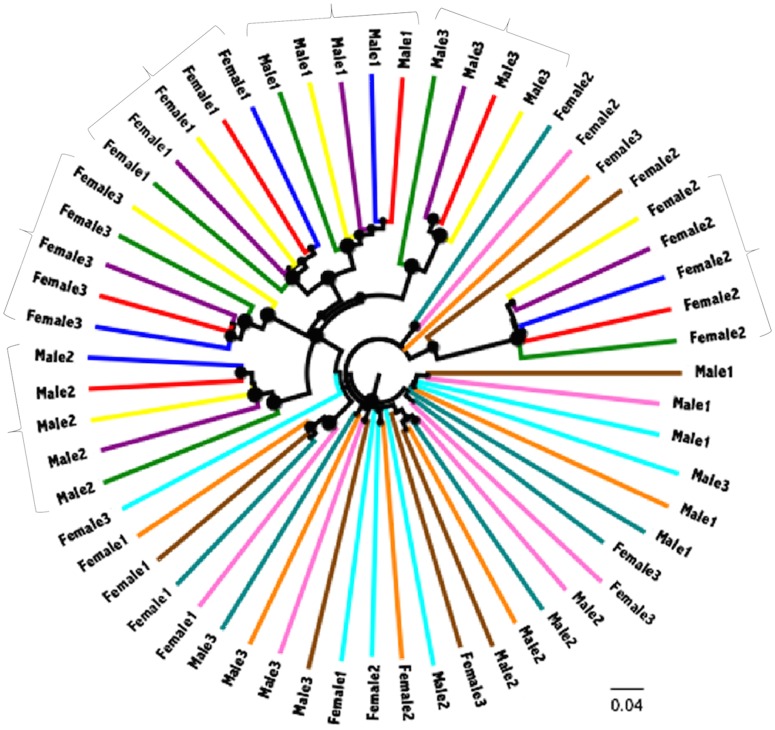
Microbial community membership is strongly associated with host individual in the lower GI tract. Tree is based on UPGMA clustering of unweighted UniFrac distance. Different colors show different gut segments (see Fig 3A). Larger node sizes indicate stronger jackknife support. The brackets show the clustering by individuals in the lower GI tract with a jackknife support of 1.0.

The stronger effect of individuals on microbial variation in the lower GI tract is further supported by UPGMA trees of community membership, where the samples (cecum to feces) from the same individual were each clustered with a jackknife support of 1.0, unlike the pattern seen in samples from the upper GI tract (Fig 4). Although the lower GI tract samples from the same individual did not always form a clade when the taxa were weighted by relative abundances, the UPGMA tree based on community structure also showed a similar trend (Figure D in S1 File). Samples collected from the same geographic site grouped individuals in some cases (Female 1 and Male 1) but not in others (Female 2 and Male 2) (Fig 4). Although the sample size is very small, there seemed to be no obvious associations between geographic site and diet measures in the current dataset (Figure E in S1 File).

## Discussion

We characterized the microbial communities from 10 locations along the GI tract in wild-caught house mice. We evaluated the relative importance of gut segment, individual host, and diet (as reflected in stable isotope measurements) in shaping differences in microbial composition. We found significant differences in microbial composition both among individuals and among gut segments. The effect of gut segments was most pronounced between the upper and lower GI tract. When these major divisions were considered separately, individual gut segments within each major division showed few differences, and differences among individual hosts showed the strongest effects on microbial composition. This suggests that individual-specific communities exist within both the upper and the lower GI tract. Below we discuss potential mechanisms underlying differences in microbial composition along the GI tract.

Observed differences in the relative abundance of anaerobic bacteria between the upper and lower GI tract were consistent with microbial oxygen requirements in humans [41] and in lab mice [12]. Oxygen concentrations show a clinal decrease from the proximal to the distal GI tract in mice [42]. The observed distribution of anaerobic microbial taxa can be explained by this oxygen gradient. Microbial oxygen requirements have been used as an explanation of microbial distribution in mucosal and luminal samples in mice, humans, and macaques [19, 43, 44]. However, we observed discrete shifts in the relative abundances of anaerobic taxa along the GI tract despite the gradual decrease in oxygen concentration.

Gut anatomy may help explain the discrete shifts in the distribution of anaerobic microbes along the GI tract. For example, house mice are hindgut fermenters, and most anaerobic fermentation takes place in the cecum, a pouch separating the small and large intestines. The cecum is where we observed an increase in anaerobic taxa. Others have made similar observations in hindgut fermenting rodents [12, 14]. In contrast, woodrats are foregut fermenters characterized by a segmented stomach in which fermentation takes place. In woodrats, anaerobic taxa were abundant throughout the GI tract including the stomach [13]. Therefore, the distribution of anaerobic taxa may in part be determined by the particular anatomy associated with different kinds of fermentation chambers. It is also possible that anaerobic taxa were overrepresented in the lower GI tract compared to the upper GI tract due to differences in luminal *versus* mucosal biomass along the GI tract. Alternative explanations for the distribution of microbial taxa are certainly possible (e.g. nutritional gradient, cell densities, immunity, etc.) and these explanations are not mutually exclusive. Characterizing the biochemical environment among the gut segments will aid in understanding the factors structuring the observed microbial communities.

Microbial diversity was greater in the lower GI tract compared to the upper GI tract. This pattern is consistent with studies in flying squirrels and lab mice [12, 14] but different from humans and woodrats. In humans, mouth samples had the highest diversity [11], and in woodrats, segmented stomach samples (where foregut fermentation takes place) were as diverse as the lower GI tract samples [13]. The differences between humans and house mice could partly be explained by differences in sampling methods and by the greater sequencing depth in humans [11]. Although mouth and esophagus samples had smaller biomass compared to the rest of the GI tract in the current study, the initial biomass differences alone cannot explain the overall pattern because the rest of the gut segments were of equal weight. In rodents, the location of the fermentation chambers (the segmented stomach and/or cecum) along the GI tract might explain the diversity pattern; the small intestine shows the least diversity and the fermentation chambers show the highest diversity [12–14].

Natural selection may favor higher microbial diversity in fermentation chambers. Lu et al. (2014) suggested that the high diversity in fermentation chambers might reflect the stability-diversity relationship observed in macro-ecology [45,46]: species-rich communities in fermentation chambers may be more stable, resilient, and recover faster from disturbance. Interestingly, herbivorous mammals also have the highest gut microbial diversity compared to omnivorous and carnivorous mammals [47] suggesting the need for high microbial diversity for plant digestion. However, diversity measures should be interpreted with caution when different sample types are compared, since the sampled luminal volume or total cell numbers are likely to be different among the gut segments. Also our data cannot distinguish live cells from dead cells. Quantifying the number of cells sampled from each gut segment, and the viability of the cells will provide a better estimate of the microbial diversity across the GI tract.

Gut microbes in the lower GI tract showed a stronger pattern of individual-specific communities compared to the upper GI tract. The harsher environment in the upper GI tract (e.g. stomach acids) may filter certain bacterial taxa and potentially reduce the individual variation in the upper GI tract compared to the lower GI tract. Alternatively, individual differences in immunity in the lower GI tract might increase the individual variation in the lower GI tract compared to the upper GI tract [48]. Understanding the effects of hosts on microbial composition is challenging for several reasons. The present study is limited not only due to the small sample size, but also due to the co-variation of multiple factors including diet, geographic site captured, sex, and host genotype. Manipulative experiments in a common environment would help characterize the effect of each variable in structuring the individual differences in microbial communities along the GI tract.

Microbiota of fecal samples were similar to those from the lower GI tract. Fecal samples had a comparable relative abundance of bacterial families, diversity measures, and predicted gene functions in comparison to those from the lower GI tract. Most of the core OTUs (93.3%) found in the fecal samples were present in the lower GI tract samples. In terms of community membership and structure, fecal samples were indistinguishable from those derived from the lower GI tract. Surprisingly, the fecal samples accurately predicted the microbial community in the lower GI tract of each individual despite the fact that some individuals in this study were captured in the same geographic site and were eating a similar diet based on isotope diet measures. Although direct sampling from the gut segments of interest is ideal when possible, fecal samples are easy to collect, non-disruptive, comparable to previous studies, and are required for some longitudinal experiments. The similar community composition between the lower GI tract and feces, and the stronger effect of individuals in the lower GI tract compared to the upper GI tract support the utility of fecal sampling for studying gut microbial communities.

We characterized substantial heterogeneity among segments of the GI tract in wild house mice. However, individual hosts also play a significant role in structuring microbial communities within particular segments of the GI tract. Further research is required to understand the specific factors affecting the microbial community composition among gut segments and among individuals.

## Supporting Information

S1 File Supplemental information. The file includes supplemental Tables A-G and Figures A-E.
(XLSX)
